# Nurses' Perspectives on the Use of Prophylactic Dressings to Prevent Pressure Injury: A Qualitative Study

**DOI:** 10.1111/jocn.17595

**Published:** 2024-12-09

**Authors:** Jake McMahon, Elizabeth McInnes, Ching Shan Wan, Nicola Straiton, Louisa Lam, Jane Rodgers, Paul Fulbrook

**Affiliations:** ^1^ School of Nursing, Midwifery and Paramedicine, Australian Catholic University Melbourne Victoria Australia; ^2^ St Vincent's Hospital Melbourne Fitzroy Victoria Australia; ^3^ Nursing Research Institute, St Vincent's Health Network Sydney, St Vincent's Hospital Melbourne, Australian Catholic University Sydney New South Wales Australia; ^4^ National Health and Medical Research Council Centre of Research Excellence in Wiser Wound Care, Griffith University Gold Coast Queensland Australia; ^5^ Respiratory Research@Alfred, School of Translational Medicine, Monash University Melbourne Victoria Australia; ^6^ St Vincent's Health Network Sydney Darlinghurst New South Wales Australia; ^7^ School of Public Health and Preventive Medicine, Monash University Clayton Victoria Australia; ^8^ Nursing Research and Practice Development Centre The Prince Charles Hospital, Metro North Health Brisbane Queensland Australia; ^9^ Faculty of Health Sciences, School of Therapeutic Sciences, University of the Witwatersrand Johannesburg South Africa

**Keywords:** acute care, pressure injury, prevention, prophylactic dressing, qualitative

## Abstract

**Aim:**

To understand, from a nursing perspective, factors affecting the use of prophylactic dressings to prevent pressure injuries in acute hospitalised adults.

**Background:**

Pressure injury causes harm to patients and incurs significant costs to health services. Significant emphasis is placed on their prevention. Relatively recently, prophylactic dressings have been promoted to reduce pressure injury development. However, in the acute care setting, information about the clinical use of these dressing is lacking.

**Design:**

Qualitative, descriptive.

**Methods:**

Nineteen medical and surgical nurses participated. Semi‐structured interviews were conducted and transcribed verbatim. Thematic analysis was performed using an inductive approach using NVivo software.

**Results:**

Three themes were identified, reflecting factors that influenced and perpetuated indiscriminate use of prophylactic dressings: *False sense of security*; *Convenience and task prioritisation*; and *Navigating challenges in evidence‐based pressure injury prevention.*

**Conclusions:**

The findings indicate inconsistent prevention practices, with prophylactic dressings often applied without justification or referral to research‐based evidence to guide clinical decision‐making. There was a prevailing attitude of ‘job done’ when a prophylactic dressing was applied.

**Impact:**

This study has identified several factors that perpetuate the inappropriate use of prophylactic dressings for pressure injury prevention that may be amenable to organisational change. The findings indicate that nurses often rely on these dressings as a shortcut due to time constraints, which led to missed skin assessments and low‐value care. The research can be used to inform the development of clear guidelines on dressings within hospital settings which encourage assessment‐based selection for their use, and process‐based guidance for their application, skin surveillance, dressing inspection and removal.

**Reporting Method:**

The Consolidated Criteria for Reporting Qualitative Research (COREQ) reporting guideline was followed.

**Patient or Public Contribution:**

Neither patients nor the public were directly involved in this study.

**Summary**

- Prophylactic dressings are used widely in clinical practice to reduce pressure injuries. However, this study has revealed a gap between real world practice and evidence of effectiveness.
- Workforce and workflow demands of clinical practice, as well as the lack of a clinical decision‐making framework to guide practice, have resulted in the indiscriminate use of such dressings.
- Use of a framework which focuses on the context of implementation would facilitate a more nuanced understanding of the environmental, social and organisation factors that influence the use of prophylactic dressings.

## 1 Introduction

Pressure injuries are defined as an *‘area of localised damage to the skin and underlying tissue caused by friction or shear and/or a combination of these’* (European Pressure Ulcer Advisory Panel, National Pressure Injuiry Advisory Panel, Pan Pacific Pressure Injury Alliance 2019). They are potentially avoidable and are among the costliest adverse events that occur in acute hospital settings (Fernando‐Canavan et al. 2021). Pressure injuries cause pain, reduce quality of life increase hospital length of stay and increase treatment costs to health services (McCosker et al. 2019). In Australia, hospital‐acquired pressure injury incidence is one of several key governmental indicators of the quality of patient care (Australian Commission on Safety and Quality in Health Care 2022; Oner et al. 2021) with treatment costs estimated to be AUD 3.59 billion in 2020, not accounting for indirect costs (Nghiem et al. 2022). Furthermore, pressure injuries were estimated to be associated with 6701 healthy years lost due to pain, reduced quality of life and premature death (Nghiem et al. 2022). Similarly, estimations of cost for hospital‐acquired pressure injuries in the United States could exceed $26.8 billion (Padula and Delarmente 2019). Globally, the incidence of hospital‐acquired pressure injuries was estimated to be 8.5% (95% CI 7.6–9.3), with the most frequently affected anatomical locations being the sacrum, followed by heel and hips (Li et al. 2020).

In general, in acute hospital settings, nurses are responsible for the prevention of pressure injuries (Li et al. 2022). Following individualised risk assessment, preventative interventions should be implemented to mitigate the risk of pressure injury development (Fulbrook, Mbuzi, and Miles 2019). Pressure injury prevention should be informed by a comprehensive risk assessment that includes an individual's risk factors. Non‐modifiable risk factors for pressure injuries include factors such as age and gender, whereas modifiable factors encompass nutritional status, mobility/activity level, skin status, moisture, perfusion, oxygenation and co‐morbid conditions (European Pressure Ulcer Advisory Panel, National Pressure Injuiry Advisory Panel, Pan Pacific Pressure Injury Alliance 2019; Wang et al. 2024). In terms of prevention, prophylactic multilayered foam dressings are used on body sites that are at high‐risk of pressure injury, such as the heels and sacrum, and are recommended in the international guideline for those at risk (European Pressure Ulcer Advisory Panel, National Pressure Injuiry Advisory Panel, Pan Pacific Pressure Injury Alliance 2019). They are widely used in the clinical setting due to their convenience, quick application and potential cost‐effectiveness (Beeckman et al. 2021; Santamaria et al. 2015). However, the evidence to support their clinical effectiveness in general hospital settings is still evolving (Walker et al. 2023).

Systematic reviews of prophylactic dressings have predominantly included studies conducted in specialised settings such as intensive care units, emergency departments and orthopaedic wards, with limited focus on acute medical and surgical wards (Fulbrook, Mbuzi, and Miles 2019; Lovegrove et al. 2021; Rahman‐Synthia et al. 2023; Sillmon et al. 2021; Sugrue et al. 2023). While meta‐analyses suggest some effectiveness (Fulbrook, Mbuzi, and Miles 2019; Sugrue et al. 2023), particularly for sacral dressings (Sugrue et al. 2023), the findings are based on studies involving high‐risk patients identified through low Braden Scale scores. Recent systematic reviews including those by Sugrue et al. (2023) and Rahman‐Synthia et al. (2023) found favourable effects for silicone dressings in preventing pressure injuries, with relative risk reduction ranging from 0.30 to 0.40. However, these studies included significant proportion of participants from intensive care settings.

Notably, randomised trials by Beeckman et al. (2021) and Oe et al. (2019) demonstrated significant reductions in pressure injuries using sacral dressings among high‐risk hospitalised patients, but these findings cannot be broadly generalised across all acute care settings. The current body of research suggests that prophylactic dressings may be beneficial for preventing pressure injuries, but their effectiveness is most evident when applied to high‐risk patients in specific clinical settings.

Despite the lack of robust supporting evidence, the application of foam dressings to the heels and sacrum to prevent pressure injury in the general medical‐surgical setting is common practice. While these interventions may benefit some patients, their use without clear justification raises questions about clinical appropriateness and the indiscriminate use of such dressings may potentially divert resources from other effective preventative measures. In the acute hospital setting of this study, in general medical and surgical wards, informal reports from nursing staff and clinical practice observations indicated that the use of prophylactic foam dressings to prevent pressure injuries was inconsistent. Clinical practice reports indicated that nurses were applying prophylactic dressings without consideration of individual patient risk, with many low‐risk patients receiving the intervention unnecessarily as a consequence. Furthermore, once placed on the patient, dressings were not being lifted regularly to enable visual assessment of the skin underneath. It is important to note that pressure injury risk scores should be used in conjunction with clinical judgement to inform care decisions, with individual risk factors taken into consideration, rather than relying on scores alone to drive prevention practices. This approach ensures appropriate interventions are put in place. To enhance the quality and efficiency of care, it is essential to understand the factors driving inappropriate or unnecessary use of interventions. This is particularly relevant in general acute ward settings, where there is a lack of research evidence to guide the use of prophylactic dressings. By examining the context of the problem and considering individual perspectives on what influences these practices, healthcare professionals can develop effective strategies to enhance behavioural change. Against this background, the following research question was developed: What are nurses' perspectives on the use of prophylactic dressings for pressure injury prevention in general medical/surgical patients, including their reasons, awareness of evidence, perceived advantages and disadvantages, and evaluation of effectiveness?

## 2 Methods

### 2.1 Aim

The aim of this study was to understand medical and surgical nurses' perspectives of factors that influence the use of prophylactic dressings to prevent pressure injuries in acute hospitalised adults.

### 2.2 Design

To address the research aim, a descriptive qualitative study using semi‐structured interviews was designed. This approach is particularly effective for obtaining detailed, direct insights into a phenomenon, especially when existing information is scarce (Turale 2020). The COnsolidated criteria for REporting Qualitative research (COREQ) reporting guideline were followed (Tong, Sainsbury, and Craig 2007). See Data S1.

### 2.3 Setting and Participants

This study was undertaken in three medical and two surgical wards in two public metropolitan hospitals in two Australian states. Nurses' participation was approved by each hospital's nursing executive, and ethical approval was obtained from both hospital and university Human Research Ethics Committees (reference: LRR140/23 & 2023‐3303R).

Nurses were eligible to participate if they met the following criteria: registered for at least 6 months as a ward‐based registered nurse or nurse unit manager; employed (full time or part time) in one of the study wards for at least 1 month, or were nurse educators or wound clinical nurse consultants who had been employed (full time or part time) in the participating acute hospital setting for at least 1 month. The inclusion of nurse unit managers, nurse educators and clinical nurse consultants was important as these roles significantly influence clinical practice and decision‐making regarding pressure injury prevention. Enrolled nurses and registered nurses that were employed on a casual basis or for less than 6 months were not included.

To ensure a variety of perspectives was obtained, a purposeful sampling approach (Palinkas et al. 2015) was used to recruit participants. Initially, nurse unit managers were asked to nominate nurses who met eligibility criteria and were agreeable to be contacted as potential participants. In addition, posters inviting nurses to participate were placed in participating wards. Potential participants were provided with a participant information sheet and consent form if they expressed interest to participate in the study. Individual interview times were arranged for consenting participants. Recruitment was informed by information power and data sufficiency and considerations about the study aim, sample specificity, theoretical background, quality of dialogue and analysis strategy all informed sufficiency of the data. Once issues had begun to be repeated and no further themes were apparent, two more interviews were undertaken to confirm that data sufficiency had been achieved.

### 2.4 Data Collection

Individual interviews were conducted either in‐person or via online video conferencing. Face‐to‐face interviews were conducted in an area away from other staff and patients to ensure participants' comfort and maintain confidentiality. The interviews were digitally audio‐recorded and, where video conferencing was used, only the audio files were saved. All interviews were conducted by a single interviewer, who was an experienced nurse, trained in qualitative interviewing. The interviews were conducted between September 2023 and April 2024. The semi‐structured interview guide was piloted with several clinical staff and researchers and minor modifications made before use. A single interview guide was developed, with a slight variation used for ward‐based clinical nursing staff to differentiate from participants not involved in bedside patient care. It comprised of open‐ended questions, probes and prompts designed to explore nurses' roles and decision‐making processes around pressure injury prevention interventions, and their experiences and views of the use of prophylactic dressings. Examples of open‐ended questions used included, ‘What are your roles and responsibilities in pressure injury prevention?’ and ‘How do you decide which patients should have prophylactic dressings applied to prevent pressure injuries?’. Prior to commencing the interview, participants were asked to complete a short survey to capture demographic information about their years of experience, current professional role, level of education and pressure injury education and practice. Brief notes were taken by the interviewer during the interviews, and more detailed descriptions of context and reflections were recorded immediately following each interview. The interviews were transcribed verbatim. All data were de‐identified, with a unique code given to each participant.

### 2.5 Data Analysis

Data were analysed using an inductive reflexive thematic analysis approach following Braun and Clarke (2022). This approach was deemed appropriate for this study as it is suitable for research that aims to develop insights into complex phenomena (Braun and Clarke 2022). Initially, the interviewer familiarised themself with the data through repeated reviews of the transcripts, thereby gaining a comprehensive understanding. NVivo software was used to organise the interview data, code the data and construct themes (QSR International Pty Ltd 2019). The entire dataset was coded by the primary researcher, assigning concise descriptive or interpretive labels for data items. This was followed by aggregation of similar codes into preliminary themes, focusing on those that directly addressed the research questions while discarding less relevant themes. Themes were then reviewed and refined through both internal consistency checks and external discussions with the research team. This process involved presenting the preliminary themes and supporting data to the team, discussion interpretations and refining the thematic structure based on collective input. After achieving a robust definition of themes that provided insightful interpretations, the final phase involved the thematic narrative being comprehensively written up in the report format (Braun and Clarke 2022).

### 2.6 Rigour and Reflexivity

The research team comprised clinicians and researchers with expertise in nursing, qualitative research methods, implementation science and pressure injury prevention and management. The interviewer (male) had previously worked as a registered nurse in one of the participating hospitals, but not on any of the participating wards, and was unknown to most participants. Data analysis was led by the interviewer, with reference to a qualitative diary that was maintained throughout data collection and analysis to document experiences, thoughts, feelings, and was guided by regular research team discussions to ensure consistency of data interpretation. As well, the diary provided a reflective record of the interviewer's experiences, allowing for ongoing self‐assessment and transparency throughout the research process. Participants were reapproached for clarification when needed, allowing for validation of interpretations and a more comprehensive capture of their perspectives.

## 3 Results

### 3.1 Participant Characteristics

There were 19 participants: five nurse unit managers, two clinical nurse educators, three clinical nurse consultants and nine ward‐based registered nurses. Their characteristics are summarised in Table 1. Interviews lasted for between 22 and 72 min.

**TABLE 1:** Participant demographics (*n* = 19).

Demographic information	Number of participants
**Nursing specialty**	
Medical	8
Surgical	11
**Hospital site**	
Melbourne	8
Sydney	11
**Gender**	
Female	16
Male	3
**Highest qualification**	
Bachelor's degree	10
Graduate certificate	7
Graduate diploma	1
Master's degree	1
**Years' nursing experience**	
0.5–2	4
2–5	3
5–10	6
10–15	2
> 15	4

### 3.2 Themes

Three main themes were identified from the analysis: *False sense of security*; *Convenience and task prioritisation*; and *Navigating challenges in evidence‐based pressure injury prevention*. These themes, summarised in Table 2, collectively, describe participants' perspectives of influences and factors that they considered in their use of prophylactic dressings. The findings are described below, using verbatim quotes from participants to illustrate the themes.

**TABLE 2:** Themes and subthemes.

Theme	Subtheme
False sense of security	Habitual dressing use Dressing induced complacency
Convenience and task prioritisation	Quick prevention fix Limited prioritisation of fundamental care
Navigating challenges in evidence‐based pressure injury prevention	Reliance on tacit knowledge Difficulties in sustaining best practice

#### 3.2.1 False Sense of Security

Reflecting on their own practices in the acute hospital setting, ward nurses acknowledged feeling reassured when applying dressings to patients, even when the risk of pressure injury was not imminent. They believed that dressing application was a proactive step in pressure injury prevention. However, subsequent discussions with participants highlighted that nurses tended to overestimate the effectiveness of dressings as a singular prevention strategy. This overreliance often led to misplaced confidence in dressings as the major pressure injury prevention strategy and meant that participants minimised the importance of other preventative measures. The subthemes *Habitual dressing use* and *Dressing induced complacency* further describe the risks of indiscriminate and inappropriate use and false reassurance given by dressings.

##### 3.2.1.1 Habitual Dressing Use

Participants raised concerns about the routine, sometimes automatic application of dressings on their ward as, despite manufacturers' guidelines to inspect the skin daily under each dressing (the dressings are designed to be taken down daily) the practice of applying a dressing discouraged daily skin inspections. As described commonly across all participant groups, there was a prevailing tendency towards applying dressings based on superficial assessments, rather than comprehensive individual assessment of patient needs. Dressings were often applied immediately upon sight of the slightest indication of skin discolouration without conducting more detailed assessments. One participant remarked:

I think they do try and do a lot of the dressings. That's a big thing as soon as they see that little bit of redness, they put a dressing on and then you find it's been there for days and hasn't been looked at because it's just covering up what's not wanting to be seen. (RN002)

When asked about the criteria for applying prophylactic dressing, one surgical registered nurse who had been nursing for around two of years explained:

Yeah, so usually patients who are at higher risk of developing a pressure sore, so like people who are not able to reposition themselves frequently, we usually just pop them on the prophylactic dressings. (RN006)

Once applied, these dressings often remained unchecked for extended periods, especially when they appeared dry and intact from the outside. Some nurses with years of experience reported that superficial assessments can mask the development of more severe pressure injuries, particularly in high‐risk areas, like the sacrum and heels, where small unnoticed deteriorations can quickly escalate.

Another registered nurse highlighted this issue, stating:

I would say that people typically just slap a dressing on and call it a day. Then potentially that [brand name] dressing hasn't been checked for several days and we don't really know what's under it. (RN009)

One participant emphasised how the habitual use of dressings, even when unnecessary, undermined continuous assessment of patients' skin integrity. Once a dressing was applied, there was a tendency to disregard what lay beneath. This aligned with a clinical nurse consultant's observation regarding a misconception among some nurses regarding the efficacy and maintenance of prophylactic dressings. Even though the clinical nurse consultant was aware that the overuse of dressing can lead to complacency and inadequate monitoring, this understanding was not shared by all nurses, as illustrated by the following account:

They don't touch them, because they believe, through the literature… and when you Google them and stuff, that they can stay on for a week. So, people say, right, not looking at it for a week. Then after a week they go and have a look at it, and because this patient is compromised, then there's a big injury underneath. (CNC001)

##### 3.2.1.2 Dressing Induced Complacency

Several participants reported that their decision to use dressings was primarily motivated by a belief in the dressing's effectiveness to prevent pressure injuries. There was notable variation in opinion regarding which patients would benefit from this intervention. Some nurses believed the dressings would benefit all patients, regardless of their individual risk of pressure injury. In contrast, others thought the primary benefit was specific to for high‐risk patients. A common theme among participants was the assumption that dressing application could significantly reduce or potentially eliminate the need for additional preventative strategies. Participants described several practices that stemmed from this belief. These included infrequent re‐assessment of dressings once applied, reduced implementation of other preventative measures, and instances where developing pressure injuries beneath dressings were overlooked. These practices were prevalent among many participants reflecting a widespread misconception about the role and efficacy of prophylactic dressings.

Despite awareness among some senior staff of the risks associated with overreliance on dressings, the practice persisted. This situation appeared to be compounded by less experienced nurses' lacking confidence in their assessment skills, leading them to over‐rely on dressings as a perceived ‘safe’ option. This behaviour was illustrated by one registered nurse who stated:

You find you put them on, you won't see what's under them because no one will bother to look because they'll think okay it's there to prevent pressure injuries, or there is already one that we know about. They probably wouldn't think to check and see if it's worsening because there is a dressing there to stop it. (RN002)

Another participant highlighted the perception that once a dressing was in place, the wound or high‐risk site appeared taken care of, leading to a lack of further intervention. The participant, a nurse educator, suggested that within their context, high‐risk patients were primarily the ones who would receive prophylactic dressings; however, the variance was, ‘depending on the individual and the education they had received’, commenting further:

I think maybe something in their minds is like the wound is taken care of by the dressing and they might not need to do anything because it looks like it's dry and intact. So, they kind of just leave it there and they say it's fine. (E001)

Some other nurses, with less experience, noted a discrepancy in practice where bedside nurses appeared to be under the misconception that once a dressing was applied, other pressure injury prevention strategies, such as regular re‐assessment, became less necessary, contrary to recommendations from dressing manufacturers. This was summarised by a surgical nurse who described the act of placing a dressing on a patient as a ‘job done’:

If they're putting them on, and there's no pressure injury, then it's that people aren't probably looking at that as regularly as they should be. I guess, once it's on everyone's like, ‘oh, okay then that's all’ and people probably think ‘Oh well that's it, job done’. You know you don't have to roll your patient because they've got a dressing in situ… It might be not be a pressure injury when you put it on, but now it's the stage two, but no one's documented that because there's just been a dressing left on for two days. (RN001)

Some nurse unit managers and clinical nurse consultants reported that despite initially using dressings prophylactically, they observed failures to complete necessary re‐assessments. This led to an increase in the incidence of pressure injuries. Reflecting on this issue, a nurse unit manager stated:

People would keep the dressing on and not actually look under it and then that's when our prevention turned into a stage one or a stage two of pressure injuries… we did have a lot of pressure injuries develop because we were using prophylactic dressings. (M005)

#### 3.2.2 Convenience and Task Prioritisation

Nurses identified reasons why prophylactic dressings were inappropriately or indiscriminately used, shedding light on how these practices had become entrenched in medical and surgical settings. Nurses resorted to using these dressings as quick and convenient solutions to prevent pressure injuries amidst competing demands, a practice described in the subtheme *Quick fix to prevention*. This entrenched practice can also lead to oversights in essential care, reflecting a task‐orientated workload approach, as described in the subtheme *Limited prioritisation of fundamental care*.

##### 3.2.2.1 Quick Fix to Prevention

Many nurses reported that pressure injury prevention, like other nursing tasks, competes with multiple responsibilities, prompting them to resort to quick measures, deemed better than nothing. This approach, which involves using prophylactic dressings, was perceived as requiring less manual labour while still demonstrating a commitment to prevention.

Reflecting on this issue, a surgical clinical nurse consultant highlighted the problematic aspect of this practice, particularly in the context of preventing the progression from blanching erythema to stage 1 pressure injury. Despite the apparent efficiency of quickly applying dressings to manage workload, this approach can lead to neglect in regular re‐assessment, potentially resulting in worsened patient outcomes. The clinical nurse consultant emphasised the need for a more thorough approach to pressure injury prevention:

Everybody likes a quick fix. If they've got an [pressure] area, they think. ‘What am I going to do? I'm going to go to that dressing that I know, I'll just go and get that and fix it up’. Then I don't have to look at it again. (Surgical CNC)

A few registered nurse participants highlighted the utility of dressings as visual cues, signalling a high‐risk patient for the development of pressure injuries. As articulated by a ward‐based registered nurse, the presence of dressings serves as a tangible reminder that fosters attentiveness among nursing staff. The nurse observed that documenting on the handover sheet and discussing the dressing during handover practices encouraged some nurses to pay closer attention to the condition of the patient's skin, leading to higher vigilance for high‐risk patients:

We've put it on the handover sheet ‘they've got a dressing’ so that the nurses will know to get in there and have a look at it whereas you know if someone doesn't have a [dressing] they tend to just go, oh, yeah, they're ambulant independent and don't tend to go looking at their skin. (RN008)

##### 3.2.2.2 Limited Prioritisation of Fundamental Care

Participants discussed how nursing assessment and pressure injury prevention were not prioritised over other responsibilities This was particularly noted among junior staff facing time constraints related to medication rounds, general observations and blood collection. The priority given to pressure injury prevention varied across different wards, with ward‐based registered nurses often mirroring the views of their nurse unit managers on the importance of these tasks.

One participant noted that with numerous tasks to manage, staff often needed to list and check off items throughout the day. Pressure area assessments, viewed as tasks that could be done at any time, were often overlooked or were found to fall off the list of tasks. This sentiment was echoed by a nurse unit manager who referred to pressure injury prevention as part of a group of nursing tasks called ‘little things’:

With juniors, I think, they're just trying to keep on top of their vitals and do their obs, and do their meds, and do this and do that, that they miss those little things still. It's not sometimes a priority for people when they're so busy. It's not in the forefront of getting all this ticked off their list for the day. (M002)

Several participants expressed that this perception of pressure area assessment as non‐urgent tasks contributed to their inconsistent practice, especially for patients at high‐risk. Some staff noted the need for more structured approaches to ensure consistent performance of these assessments. Ward‐based registered nurse also addressed that skin assessments were often done opportunistically, regardless of the patient's risk level for pressure injuries. It was seen as something that could be done when completing another task such as changing an incontinence pad which makes it difficult to prioritise skin assessments underneath dressings. One nurse described the situation:

If they're not incontinent or things like that, then it's very rare that people will roll them over unless you're doing those regular pad changes or things like that, is what I've noticed. (RN005)

However, this view contrasted with the perspective of some nurse unit managers, highlighting a significant mismatch between nurse unit managers expectations and clinical staff practices:

We do daily skin assessments. So, every patient, well, not every patient, but if they're a moderate to high risk, which is 99.9% of our patients, need daily skin assessments and that's documented on the form. (M001)

Ward‐based registered nurses reported that once a prophylactic dressing was in place there was limited time to take it down and assess the underlying skin, citing time constraints and competing priorities. This practice was reported to occur even for patients considered at high‐risk for pressure injuries. Some nurses indicated that it was easier to deprioritise pressure injury prevention compared to other responsibilities that would be immediately noticed, such as to ‘*not give a medication’*. One nurse educator explained:

I think staff would always prioritise the obs and medication of a patient, because I think that's super time constricted for the patient, it would be 8:00, 12:00, blah, blah, blah but with the wounds and stuff like that, the afternoon shift can actually check that wound instead of me (E002)

This attitude to deprioritise pressure injury prevention was supported by some nurse unit managers who viewed pressure injury prevention as less urgent compared to other tasks such as vital sign assessments and medication administration. Although nurses expressed concerns about patients' pressure injury risk, they prioritised their activities based on urgency. For instance, they focused on routine vital sign monitoring, which might require immediate medical intervention, rather than prioritising pressure injury prevention. This practice extended to patients considered at high risk for pressure injuries. One nurse unit manager explained:

Being able to time manage all the things we expect them to do, including hygiene and looking at pressure injuries, that comes a little bit later. It's not, I suppose, not as important to us right at the start… We're a lot more worried about those time critical tasks than we are for those not time critical tasks. (M005)

#### 3.2.3 Navigating Challenges in Evidence‐Based Pressure Injury Prevention

In addressing pressure injury prevention, participants stated that they often relied on intuition rather than research, preferring guidance from senior colleagues over reviewing hospital guidelines (*Reliance on tacit knowledge*). Participants expressed openness to education, noting a lack of recent emphasis on prevention and a high turnover of staff, posing challenges to maintaining up‐to‐date practices and achieving widespread knowledge translation (*Difficulties in sustaining best practice*).

##### 3.2.3.1 Reliance on Tacit Knowledge

Nurses' understanding of pressure injury prevention strategies appeared to stem from ingrained practices rather than formal education or current research. Many participants admitted to not consulting hospital policies, instead relying on internalised collectively reinforced tacit knowledge. This knowledge was often gained through brief readings of policies and procedures, and more heavily through discussions with colleagues. While this approach was consistent throughout the interviews, clinical nurse consultants did not share this perspective. One nurse unit manager discussed how nurses felt like they knew what to do, bypassing formal policies:

I've never looked at the pressure injury prevention policy ever. Because we kind of just feel like we know what there is to do. (M003)

This sentiment was echoed by a registered ward nurse, who highlighted the reliance on verbal transmission of practices from more experienced colleagues, rather than direct engagement with the latest research and policy updates:

I don't know, to be honest. I haven't sat around and read up on it. I can only tell you that I've been advised throughout my nursing career. It's always been the next senior nurse, said dressings. You put them on it. That's what we've been told to do. (RN002)

Multiple participants expressed similar views, indicating a widespread practice of relying on verbal guidance from senior staff rather than consulting written policies or research evidence.

##### 3.2.3.2 Difficulties in Sustaining Best Practice

With the recent pandemic, high staff turnover (in part due to the COVID‐19 pandemic impact on nursing workforces) has led to a predominately junior workforce. This situation has amplified the need to familiarise new staff members with established practices. Participants noted a decrease in emphasis on updating practices with the latest evidence due to the hospital's focus on addressing staffing shortages. One ward registered nurse acknowledged a junior workforce meant there was less priority given to regular evidence updates:

I haven't been to an in‐service [education session] for pressure injuries for a while… It's good to go to them regularly because there's obviously new research, there's new ways coming out to prevent pressure injuries. (RN003)

Another nurse unit manager highlighted the importance of having multiple strategies to reach nursing staff due to logistical challenges with education sessions:

I don't know if people are properly educated about it. This again, this turnover of staff. Not everyone would know. I said I can't remember the last time I've had a pressure injury prevention [education session]. If you have an education session, let's say today you're only getting a handful of staff. Maybe they can't all come into the session, but what about, you know, every other day? Not everyone gets the training, you know. (M003)

Senior nurse participants emphasised the need to realign staff knowledge with current evidence, particularly by reviving practices that had been effective before the pandemic. They discussed the previous implementation of ward‐based ‘champions’—both junior and senior—which had been a successful strategy but had lapsed during the COVID‐19 crisis. One clinical nurse consultant reflected on this past practice and potential reinstatement:

I think having resource persons on the ward. So maybe a skin champion, someone that the junior staff can go to. We want to have a senior staff member and a junior staff member. I think that would be really helpful. They're obviously not there all the time. But that would be a resource person that does shift work, so would be around (CNC002)

The challenge of integrating evidence into practice was evident, especially when external facilitators attempted to update best practices without consideration of the ward context. This issue highlighted the complexity of translating general evidence‐based guidelines into the different environments of individual wards. A ward‐based registered nurse suggested that education alone might not be sufficient to change this behaviour:

Having a nurse synthesise the nursing considerations of all those disciplines and present it in a really clear, concise way. I feel like that would actually help in terms of changing behaviour. (RN009)

Participants reported instances of failed practice changes when allied health, medical and nursing staff from outside the ward environment attempted to implement changes without a deep understanding of the ward's dynamics. To address this issue, participants suggested regular context‐specific training sessions to consider the unique challenges of each ward. They believed this approach could enhance the relevance and uptake of best practices. Participants emphasised the importance of ensuring education considers the practical challenges of implementing prevention measures within the context of competing priorities and ward‐specific workflows.

## 4 Discussion

This study provides important insight into factors that influence the use of prophylactic dressings to prevent pressure injury in acute medical and surgical settings. The findings indicate that there was indiscriminate use of dressings, coupled with a failure to regularly assess the skin beneath them. Nurses frequently implemented these dressings as a perceived shortcut, potentially in response to competing demands and time constraints inherent in the hospital environment. This approach was perceived as a time‐saving measure that seemingly allowed staff to attend to other tasks considered to be more urgent, yet it inadvertently promoted care that was misaligned with evidence‐based practices.

A notable finding of this study was the reliance on prophylactic dressings in pressure injury prevention practices, often at the expense of regular skin assessments. While dressings played a role in pressure injury prevention, participants indicated that their use sometimes created a barrier to thorough and frequent skin checks. This practice raises concerns about the potential for missed early signs of skin damage. The emphasis on dressing use may reflect a broader issue within nursing practice where visible interventions are prioritised over less tangible preventative measures. Nurses in our study demonstrated awareness of the importance of skin assessments but described challenges in consistently implementing them alongside other care tasks perceived as more urgent. Similarly, in a recent mixed‐methods study of nurses and physicians, there was widespread recognition of ‘do not do’ recommendations in wound care that were nonetheless ignored due to entrenched habits (Verkerk et al. 2023).

In our study, nurses demonstrated a willingness to act on pressure injury prevention, yet their reported actions, such as reliance on dressing at the expense of regular skin assessments, suggest a gap in understanding of best practice. Multiple factors may contribute to this discrepancy. Nurses may have felt pressured to prioritise visible interventions over less tangible preventive measures. In a qualitative study in a critical care setting, Bourgault and Upvall (2019) found that nurses recognised low‐value practices but often lacked confidence in advocating for their change and struggled to de‐implement tradition‐based practices, and sometimes failed to distinguish between evidence‐based and tradition‐based practices. These findings suggest the need for strategies that go beyond simply providing knowledge. Future interventions focusing on creating supportive critical and reflexive thinking environments may empower nurses to question established practices, advocate for evidence‐based care and effectively integrate preventative measures into their workflow. For example, healthcare organisations could implement regular educational meetings or forums that encourage open discussions of current practices, allowing nurses to voice concerns and suggest improvements (Jeong and Kim 2023). Another approach could be establishing mentorship programs where experienced nurses guide newer staff in implementing evidence‐based practices (Hoover et al. 2020). A multifaceted approach addressing barriers at the system, organisational and individual levels may be appropriate to enhance the evidence‐based use of prophylactic dressings in pressure injury prevention, which will be evaluated in future trials.

In our study, an important finding was the consistent high prioritisation of medication administration and vital sign assessment over the prevention of pressure injuries. This prioritisation may reflect the time constraints nurses face, with these essential tasks consuming a significant portion of their available time, leaving less for important care activities. While dressings are indeed part of fundamental nursing care, our findings suggest a need for a more balanced and tailored approach to patient care. This aligns with existing evidence on missed care, as demonstrated by Cho et al. (2020) in their cross‐sectional study of nurses on general medical and surgical units. They found that activities such as turning patients two‐hourly, patients bathing/skin care and ambulation were frequently missed, while vital signs were rarely overlooked.

The consistent prioritisation of medication administration and vital signs, while crucial, may inadvertently, contributing to the neglect of essential nursing care, such as pressure injury prevention. This highlights the need for a more integrated and patient‐specific strategy. Rather than adhering to routine practices, healthcare organisations might benefit from implementing more stringent personalised assessments to determine the frequency and necessity of tasks such as vital sign measurements and medication administration. Steinman et al. (2021) described the de‐implementation of established practices, such as routine medication administration as a ‘wicked problem’ resistant to change. However, addressing these challenges is crucial to optimise patient care. In their commentary, Norton and Chambers (2020) emphasised the importance of understanding barriers and facilitators when working to reduce inappropriate health interventions. Supporting this view, in a mixed‐methods systematic review on barriers and facilitators to implementing pressure injury prevention guidelines in acute care, Wan et al. (2023) found that clinicians reported practical difficulties in adhering to these guidelines due to competing priorities and significant workload demands. This limited their capacity to engage in pressure injury assessment and prevention measures.

As found in our study, institutional pressures and a predominantly junior workforce post‐COVID‐19 have created new challenges in healthcare settings. The pandemic has led to an increase in junior nursing staff, with many experienced nurses leaving the profession due to burnout and stress (Labrague and de Los Santos 2021). This shift towards a predominantly junior workforce amplifies existing challenges in prioritising care tasks, particularly in complex areas like pressure injury prevention. These workforce challenges intersect with psychological tendencies that influence clinical decision‐making. Feldman (2020) described how the fear of inaction, especially if previously associated with negative outcomes, can drive nurses towards ineffective actions rather than optimal inaction. Action orientation, coupled with the tendency to favour additive solutions over subtractive ones, could be another critical factor influencing the misuse of prophylactic dressings. Cognitive scientists describe how people tend to search for and implement additive changes more readily than subtractive ones (Adams et al. 2021). In the context of pressure injury prevention, nurses may default to applying dressings (as additive action) rather than focusing on removing obstacles to effective skin assessment (a subtractive action). This bias towards addition can lead to the overuse of dressings, as described in this study, where the ease and visibility of applying dressings overshadows the need for comprehensive skin care.

Despite consistent industry guidance emphasising that dressings do not replace comprehensive pressure injury prevention strategies and can be used to support prevention protocols (Mölnlycke Health Care group 2018), the practice of indiscriminate and inappropriate use of dressings for pressure injury prevention in acute care wards persists. The concept of ‘mindlines’ (Gabbay and le May 2004) encapsulates the knowledge‐in‐practice‐in‐context, which often diverges from formal guidelines. The mindlines concept highlights that clinicians rarely access formal research findings and guidelines directly, instead rely on collectively internalised guidelines informed by brief readings and interactions with peers and opinion leaders (Wieringa and Greenhalgh 2015). This preference for mindlines over formal guidelines can lead to practices that diverge from evidence‐based recommendations. Our findings are consistent with this view; illustrating how nurses relied on habitual practices and peer guidance rather than updating their knowledge with the latest research.

Our findings highlight several key factors influencing nurses' clinical decision‐making regarding the application of prophylactic dressings. These include time constraints, lack of ongoing skin assessment and insufficient knowledge about the correct use of dressings for modifiable pressure injury risk factors. Notably, participants in our study raised concerns about potential issues arising from inappropriate dressing use, highlighting the need for improved education and guidelines in this area. Nurses often face barriers to accessing and implementing evidence‐based practices. For instance, in the United States, Melnyk et al. (2018) found that only 30% of nurses reported consistently implementing evidence‐based practices, and 76% felt they needed more education on the topic. The lack of accessible context‐specific training further compounds this issue, as nurses may feel ill‐equipped to challenge established routines or advocate for better practices. Grimshaw et al. (2020) noted that reducing misuse of practices or low‐value care require tailored strategies that address both individual and organisational barriers.

Nurses and other healthcare providers should be empowered to critically evaluate the use of prophylactic dressings, considering individual patient needs and the latest evidence, to ensure appropriate application and management. Using methods such as clinical audits, feedback from frontline staff and clinical decision support tools (Colla et al. 2016) can help healthcare organisations to identify and address issues such as dressing overuse, thereby promoting high‐value care and reducing unnecessary interventions. As well as developing a context‐based understanding of the issues at hand, achieving behavioural change is essential. Lavallée et al. (2019) used the Behaviour Change Wheel (Michie, van Stralen, and West 2011) to develop a pressure injury care bundle in a home care setting. They identified capability, opportunity and reflective motivation as influencing the pressure injury prevention behaviour, requiring use of education‐based interventions alongside behaviour change techniques. This theory‐informed, structured approach facilitated a thorough process for the development of the care bundle. Similar themes have emerged from our study, suggesting that this type of theory‐based approach may be effective within the study setting. Our findings also emphasised contextual issues such as competing interests and workforce and workflow issues. Using a determinant framework such as the integrated Promoting Action on Research Implementation in Health Services (i‐PARIHS) (Harvey and Kitson 2016), which focuses on the context of implementation, could help facilitate a more nuanced understanding of the environmental, social and organisation factors that influence the use of prophylactic dressings and help identify strategies to address barriers to effective practice.

### 4.1 Strengths and Limitations

This study captured multiple nursing perspectives, ranging from managers to ward‐level nurses, across two hospital sites, providing valuable insights into the use of prophylactic dressings; an area which has not previously been investigated. Its inductive approach enabled a deeper understanding of nurses' motives around the use of such dressings. Although purposive sampling ensured a good representation of participants, a greater number of surgical nurses participated, which may have influenced the findings to some extent. Although the qualitative nature of this study means that the findings are not generalisable beyond the study setting, they may represent similarities with other settings such as long‐term care and specialty settings.

### 4.2 Implications for Policy and Practice

This study has identified several potential areas for improvement that could be explored in future research. Most notable was the lack of a decision‐making framework to guide the evidence‐based implementation of prophylactic dressings. Development of a clear guideline could considerably improve practice in this area. Key elements should be focused on assessment‐based selection criteria for their use, and process‐based guidance for their application, skin surveillance, dressing inspection and removal. The importance of thorough skin assessments and individualised care plans should be emphasised, promoting a shift from the tradition‐based model to an effective evidence‐based model of care. As well, the development and evaluation of regular, context‐specific education sessions tailored to the unique challenges and workflows of each ward will help to support effective implementation. This could be supported by mentorship programs, or ward ‘champions’ whereby experienced nurses provide guidance and support to junior staff could help bridge the gap between knowledge and practice. Strategies that address both individual and organisational factors are most likely to ensure more effective high‐value nursing practices in pressure injury prevention.

## 5 Conclusion

This study adds new insights into the complex and interconnected factors that influence pressure injury prevention practices in nursing, particularly regarding the use of prophylactic dressings on general medical and surgical wards. The findings indicate that competing aspects of nursing care took precedence over pressure injury prevention practices. There was widespread and indiscriminate use of prophylactic dressings, with a lack of referral to research‐based evidence to guide clinical decision‐making. In the main, in terms of pressure injury prevention, there was a prevailing attitude of ‘job done’ when a prophylactic dressing was applied. Of further concern was the apparent lack of follow up and re‐assessment of the skin, sometimes resulting in pressure injuries.

As well as the need for a clear evidence‐based guideline to enhance clinical decision‐making, our findings indicate a need for context‐specific training, mentorship programs and accessible education to promote evidence‐based practices. Future research could explore the development of context‐specific training programs that address the unique challenges faced by nurses in different ward settings with evaluation of their effectiveness to bring about behavioural change that results in clinically effective practice. In the study setting, the findings will be used to inform the development of an evidence‐informed intervention to optimise the use of prophylactic dressing for pressure injury prevention in medical/surgical patients, and promote high‐value, evidence‐based care that enhances patient outcomes while efficiently utilising healthcare resources.

## Author Contributions

**Jake McMahon:** conceptualisation; data curation; funding acquisition; investigation; formal analysis; writing – original draft; writing – review and editing. **Elizabeth McInnes:** conceptualisation; funding acquisition; investigation; methodology; supervision; writing – review and editing. **Ching Shan Wan:** conceptualisation; funding acquisition; supervision; writing – review and editing. **Nicola Straiton:** supervision; writing – review and editing. **Louisa Lam:** supervision; writing – review and editing. **Jane Rodgers:** conceptualisation; supervision; writing – review and editing. **Paul Fulbrook:** conceptualisation; methodology; supervision; writing – review and editing.

## Conflicts of Interest

The authors declare no conflicts of interest.

## Supporting information


Data S1:

## Data Availability

The data that support the findings of this study are available on request from the corresponding author and participants. The data are not publicly available due to privacy or ethical restrictions.
